# Induction of Apoptosis Coupled to Endoplasmic Reticulum Stress in Human Prostate Cancer Cells by *n*-butylidenephthalide

**DOI:** 10.1371/journal.pone.0033742

**Published:** 2012-03-28

**Authors:** Sheng-Chun Chiu, Shee-Ping Chen, Sung-Ying Huang, Mei-Jen Wang, Shinn-Zong Lin, Horng-Jyh Harn, Cheng-Yoong Pang

**Affiliations:** 1 Department of Medical Research, Buddhist Tzu Chi General Hospital, Hualien, Taiwan; 2 Tzu Chi Stem Cells Center, Buddhist Tzu Chi General Hospital, Hualien, Taiwan; 3 Department of Ophthalmology, Mackay Memorial Hospital, Hsinchu, Taiwan; 4 Center for Neuropsychiatry, China Medical University Hospital, Taichung, Taiwan; 5 Department of Pathology, China Medical University Hospital, Taichung, Taiwan; 6 Institute of Medical Sciences, School of Medicine, Tzu Chi University, Hualien, Taiwan; Faculty of Pharmacy, Ain Shams University, Egypt

## Abstract

**Background:**

*N*-butylidenephthalide (BP) exhibits antitumor effect in a variety of cancer cell lines. The objective of this study was to obtain additional insights into the mechanisms involved in BP induced cell death in human prostate cancer cells.

**Methods/Principal Findings:**

Two human prostate cancer cell lines, PC-3 and LNCaP, were treated with BP, and subsequently evaluated for their viability and cell cycle profiles. BP caused cell cycle arrest and cell death in both cell lines. The G0/G1 phase arrest was correlated with increase levels of CDK inhibitors (p16, p21 and p27) and decrease of the checkpoint proteins. To determine the mechanisms of BP-induced growth arrest and cell death in prostate cancer cell lines, we performed a microarray study to identify alterations in gene expression induced by BP in the LNCaP cells. Several BP-induced genes, including the GADD153/CHOP, an endoplasmic reticulum stress (ER stress)-regulated gene, were identified. BP-induced ER stress was evidenced by increased expression of the downstream molecules GRP78/BiP, IRE1-α and GADD153/CHOP in both cell lines. Blockage of IRE1-α or GADD153/CHOP expression by siRNA significantly reduced BP-induced cell death in LNCaP cells. Furthermore, blockage of JNK1/2 signaling by JNK siRNA resulted in decreased expression of IRE1-α and GADD153/CHOP genes, implicating that BP-induced ER stress may be elicited via JNK1/2 signaling in prostate cancer cells. BP also suppressed LNCaP xenograft tumor growth in NOD-SCID mice. It caused 68% reduction in tumor volume after 18 days of treatment.

**Conclusions:**

Our results suggest that BP can cause G0/G1 phase arrest in prostate cancer cells and its cytotoxicity is mediated by ER stress induction. Thus, BP may serve as an anticancer agent by inducing ER stress in prostate cancer.

## Introduction

Prostate cancer is the most common malignancy in American men and the second leading cause of deaths from cancer [1]. The treatment options for patients include surgery, radiation therapy, hormonal therapy, chemotherapy, and combinations of some of these treatments. One of the most common chemotherapy agent used for the treatment of prostate cancer are the taxanes, such as the first generation drug paclitaxel (Taxol, a trademark of Bristol-Myers Squibb) [2], [3]. Increased concentrations of cytotoxic drugs and higher dosages of irradiation fail to improve the response to therapy and it leads to apoptosis resistance in prostate cancer cells. Therefore, newly developed anticancer agents that are nontoxic and highly effective in inducing apoptosis preferentially in tumor cells are valuable.

Recently, non-traditional treatments using herbs and dietary supplements have been considered as alternative medicines for cancer therapy. *Angelica sinensis* (also called danggui in Chinese) is one of the most commonly used traditional herbs in China. It is recommended as a tonic, hemopoetic, spasmolytic, and analgesic drug in clinical practice [4]. In our previous study, *n*-butylidenephthalide (BP), a compound isolated from *Angelica sinensis* chloroform extract, exhibits growth inhibitory activity on various human cancer cell lines, including brain, lung and liver cancer cells. BP caused growth arrest and apoptosis in these tumor cells *in vitro* and *in vivo* [5]–[7]. These findings indicate that BP is a promising new anticancer compound with a potential for clinical application. However, the effect of BP on prostate cancer cells has not been addressed.

Protein folding in the endoplasmic reticulum (ER) is impaired under various physical and pathological conditions, termed ER stress. A specific signaling pathway, the unfolded protein response (UPR), has been demonstrated in cell to overcome ER stress [8]. Induction of glucose-regulated protein GRP78/BiP has been widely recognized as a marker for ER stress and the onset of UPR. The UPR signals through three distinct stress sensors located at the ER membrane: protein kinase RNA-like ER kinase (PERK), inositol-requiring protein-1 α (IRE1-α) and activating transcription factor-6 (ATF6) [9]. Among them, IRE1-mediated activation of Jun N-terminal kinase (JNK) contributes to cell death by phosphorylating and inactivating the anti-apoptotic regulator BCL-2 [10]. The transcription factor CCAAT/enhancer-binding protein (C/EBP)-homologous protein (CHOP; also known as DDIT3/GADD153) operates at the convergence of the PERK, IRE1-α and ATF6 pathways [11], [12]. Overexpression of CHOP plays an important role in apoptosis [13] through dephosphorylates the proapoptotic BH3-only protein Bad and down-regulates Bcl-2 expression [14].

This present study sought: 1) to determine the anti-proliferative effect of BP *in vitro* and *in vivo*, 2) to assay the effects of BP on cell cycle progression and apoptosis, 3) to investigate ER stress as a potential molecular target of BP, and 4) to establish whether MAPK/ER stress signaling is involved in BP-mediated apoptosis in prostate cancer. The effects of BP in prostate cancer were evaluated *in vitro* in LNCaP and PC-3 cell lines. LNCaP-NOD-SCID mice xenograft experiment was used to assess the anticancer effect of BP *in vivo*.

## Results

### BP inhibited proliferation and induced morphology changes in human prostate cancer cells

**Figure 1:**
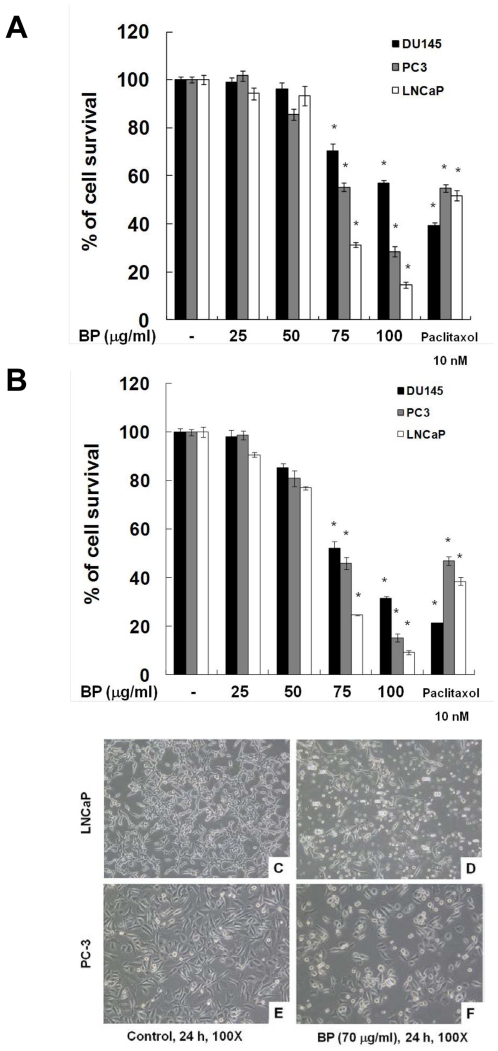
Effects of BP on the viability and morphological changes of human prostate cancer cells. DU-145 (black), PC-3 (grey), LNCaP (white) cells were treated with increasing concentration of BP (25 to 100 µg/ml) for 24 (A) and 48 h (B) and analyzed with MTT assay. LNCaP and PC-3 cells were treated with 0.2% DMSO (C and E, respectively) or 70 µg/ml BP (D and F, respectively) for 24 h, were shown.

BP has a strong anti-proliferative effect on GBM cells and caused G0/G1 phase arrest and apoptosis in a time- and concentration-dependent manner. To determine the cytotoxicity effect of BP on prostate cancer cells, three human prostate cancer cell lines were treated with increasing concentration of BP for 24 and 48 h, and were evaluated by the MTT assay. As shown in Fig. 1A and B, BP significantly decreased the viability of DU-145, PC-3 and LNCaP cells in a dose and time-dependent manner. Treatment of LNCaP and PC-3 cells with 75 µg/ml BP for 48 h resulted in 24.48% and 45.88% cell survival, respectively (Fig. 1B). Based on these data, we used 70 µg/ml BP for subsequent studies. To further investigate the cytotoxicity effect of BP on prostate cancer cells, LNCaP (androgen-dependent) and PC-3 (androgen-independent) cells were selected for further investigation. BP-treated LNCaP and PC-3 cells showed obvious cell shrinkage and rounding up, features typical of cells undergoing apoptosis (Fig. 1D and F).

### BP induced cell cycle arrest at G0/G1 phase and changed the expression levels of G0/G1 regulatory proteins

**Figure 2:**
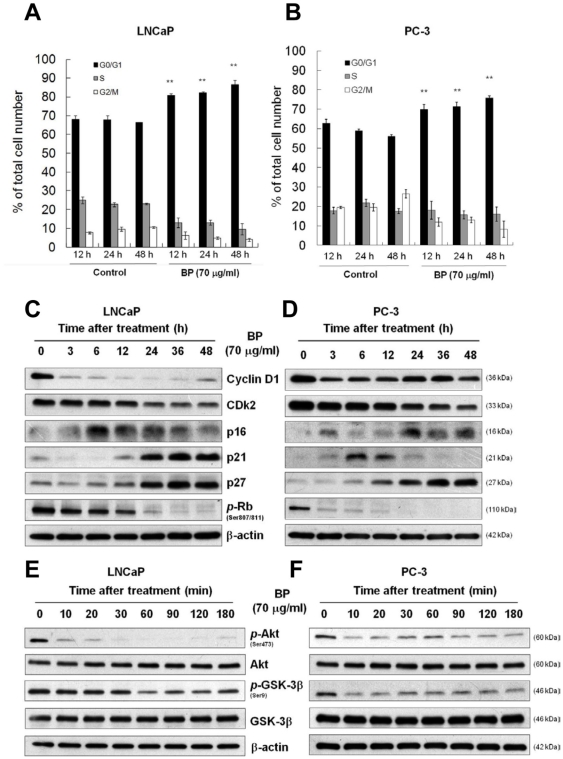
BP induces G0/G1 arrest and changes the expression profiles of G0/G1 regulatory proteins. BP induced cell cycle G0/G1 arrest in (A) LNCaP and (B) PC-3 cells. Cells were seeded at 8×10^5^ (LNCaP) and 6×10^5^ (PC-3) per 6-cm plate in triplicates and treated with 70 µg/ml BP for 12–24 h. Data are presented as means ± S.D. from three different experiments. *, *P*<0.05; **, *P*<0.01. Western blot analysis of cyclin D1, CDK2, p16, p21, p27 and phospho-Rb (Ser807/811) was performed in LNCaP cells (C) and PC-3 cells (D). β-actin was used as an internal control. Western blot analysis of phospho-Akt (Ser473), Akt, phospho-GSK-3β (Ser9) and GSK-3β was performed in LNCaP cells (E) and PC-3 cells (F). β-actin was used as an internal control.

In order to elucidate its mode of action, we examined the effects of BP on cell cycle progression. Flow cytometry analysis showed that BP treatment resulted in the accumulation of cells in G0/G1 phase in a time-dependent manner (Fig. 2A and B). Quantification of proliferating untreated cells showed that 67.95% of cells were in the G0/G1 phase, 24.96% of cells were in the S phase, and 7.73% of cells were in the G2/M phase of cell cycle 12 h after plating in LNCaP cells; 62.71% of cells were in the G0/G1 phase, 17.78% of cells were in the S phase, and 19.50% of cells were in the G2/M phase of cell cycle 12 h after plating in PC-3 cells. Treatment of cells with 70 µg/ml BP for 12 h increased the percentage of cells in the G0/G1 phase to 81.94% and reduced the percentage of the cells in the S and G2/M phases to 10.6 and 8%, respectively, in LNCaP cells. In PC-3 cells treated with 70 µg/ml BP for 12 h, the percentage of cells in the G0/G1 phase increased to 69.94%, and the cells in the S and G2/M phases decreased to 18.11 and 11.97%, respectively.

To determine the molecular mechanisms underlying the G0/G1 cell cycle arrest in prostate cancer cells induced by BP, we examined the expression of certain G0/G1 regulatory proteins in LNCaP and PC-3 cells treated with 70 µg/ml BP. We first examined the level of cyclin D1, the main cyclin controlling the G0/G1 checkpoint . BP treatment led to rapid reduction of cyclin D1 protein level (Fig. 2C and D). Upregulation of G0/G1 cell cycle regulatory proteins such as p16, p21, p27, and downregulation of CDK2 were also observed in a time-dependent manner (Fig. 2C and D).We also observed a rapid reduction of Rb phosphorylation level (Fig. 2C and D) indicating the compromised activation of the CDK4/6.

It has been established that cyclin D1 phosphorylation is mediated by the Akt-GSK-3β pathway. Activated Akt phosphorylates and inhibits GSK-3β function, leading to the de-phosphorylation and stabilization of cyclin D1. Therefore, we examined whether the Akt-GSK-3β-Cyclin D1 pathway was involved in BP-induced degradation of cyclin D1 in prostate cancer cells. Western blot analysis showed that BP rapidly and markedly suppressed Akt phosphorylation, as early as 10 min (Fig. 2E and F). Consistent with the inhibition of Akt activity, reduced phosphorylation of Ser9 in GSK-3β, a target of Akt kinase, was also noted (Fig. 2E and F), suggesting that BP promotes cyclin D1 phosphorylation via suppression of Akt and activation of GSK-3β. These data suggest that BP suppresses Akt-GSK-3β pathway in prostate cancer cells and causes G0/G1 growth arrest by affecting the expression of G0/G1 regulatory proteins.

### BP induced apoptosis in LNCaP and PC-3 cells

**Figure 3:**
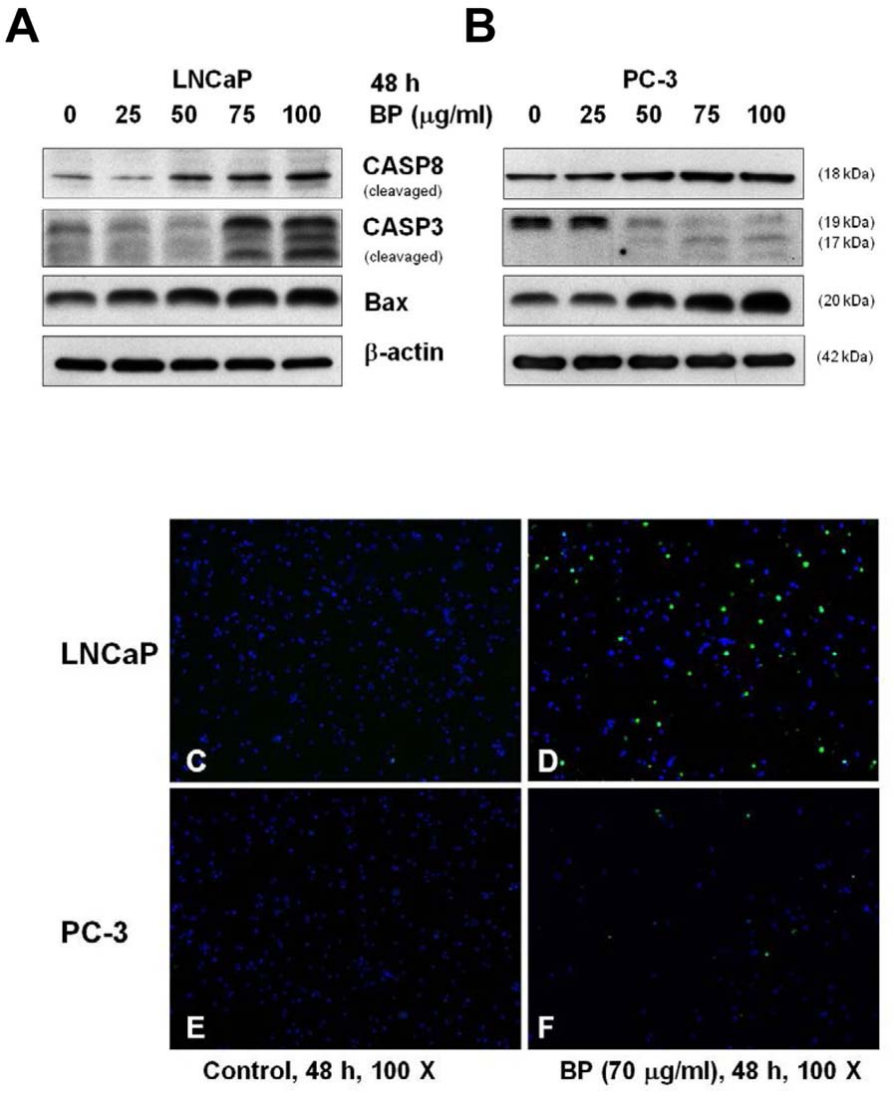
BP induces apoptosis in prostate cancer cells. Western blot analysis of active caspase 8, active caspase 3 and bax was performed in LNCaP cells (A) and PC-3 cells (B). β-actin was used as an internal control. LNCaP cells and PC-3 cells were incubated in the absence (C and E, respectively) or presence (D and F, respectively) of 70 µg/ml BP for 48 h and then subjected to TUNEL assay.

To evaluate the role of apoptosis in BP-induced cell death, western blotting and TUNEL staining were performed. Activation of caspases are crucial mechanisms for induction of apoptosis. Caspase −8 and −3 were key protease associated with death receptor mediated-apoptosis and their involvement in BP-induced apoptosis was investigated in both LNCaP and PC-3 cells. BP-induced caspase −8 and −3 cleavages increased dose-dependently in both LNCaP and PC-3 cells (Fig. 3A and B). The pro-apoptotic Bcl-2 family members, Bax, also increased after BP treatment which have important link between IRE1 and ER-stress-induced apoptosis. TUNEL staining at 48 h after 70 µg/ml BP treatment also revealed increased number of apoptotic cells in both LNCaP and PC-3 cells (Fig. 3D and F, respectively).

### Gene expression analysis by cDNA microarray in BP-treated LNCaP cells

**Figure 4:**
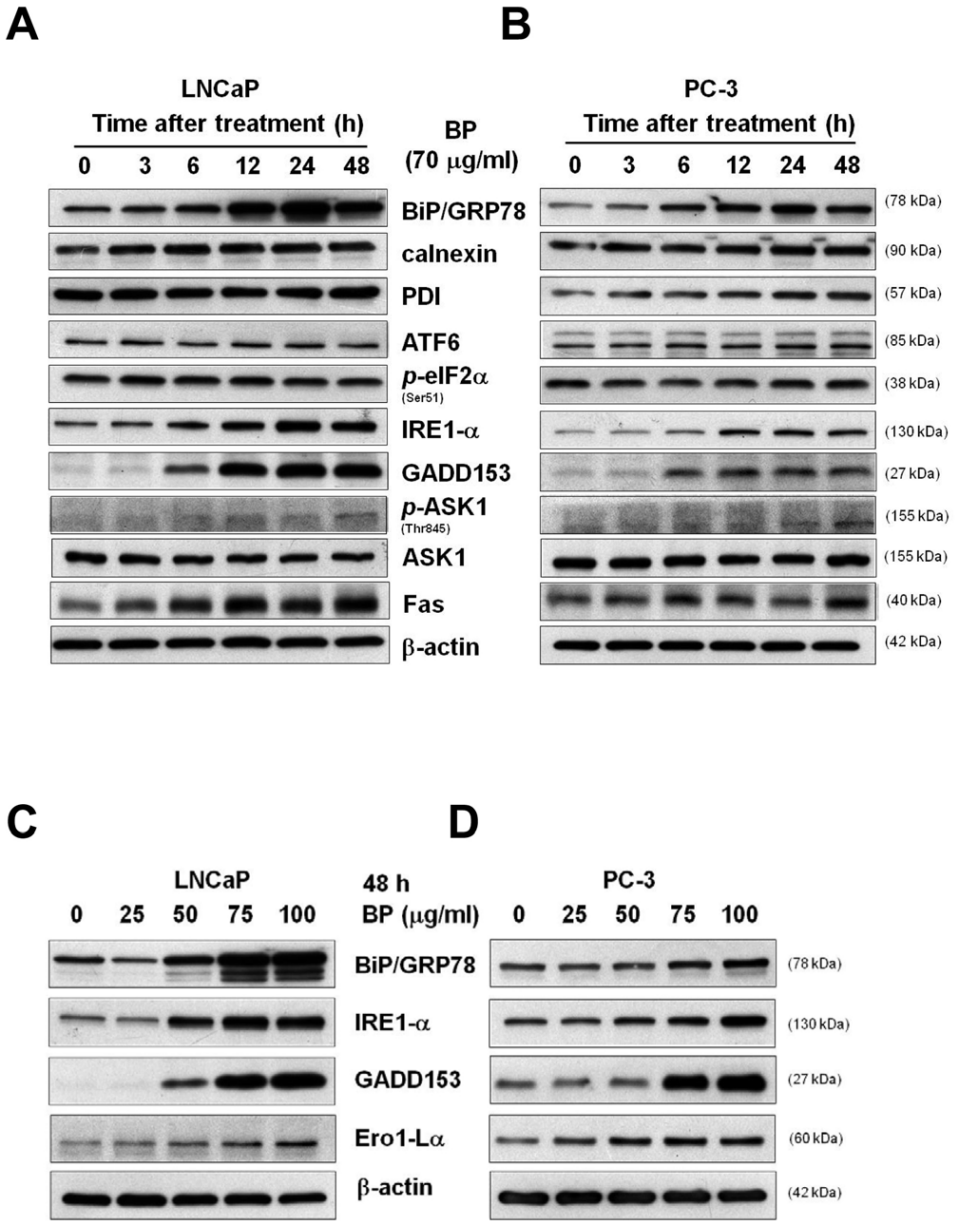
BP induces ER stress-related genes expression in prostate cancer cells. Western blot analysis of BiP, calnexin, PDI, ATF6, phospho-eIF2α (Ser51), IRE1-α, GADD153/CHOP, phosphor-ASK1 (Thr845), ASK1 and Fas were performed in LNCaP cells (A) and PC-3 cells (B). β-actin was used as an internal control. Western blot analysis of BiP, IRE1-α, GADD153/CHOP and Ero1-Lα were performed in LNCaP cells (C) and PC-3 cells (D). β-actin was used as an internal control.

To obtain insight into the mechanism of BP-induced growth inhibition and cell death, we used oligodeoxynucleotide-based microarray to identify BP-mediated gene expression. LNCaP cells were treated with 70 µg/ml BP for 3 and 24 h, and RNA was extracted and used in microarray analysis. Genes including GADD153/CHOP were identified to be up-regulated in a time-dependent manner (Table 1). We validated the up-regulation of GADD153/CHOP genes by western blot. BP elicited an increased expression of GADD153/CHOP protein in both LNCaP and PC-3 cells (Fig. 4A and B). Up-regulation of GADD153/CHOP has been defined as ER stress-response, which in turn triggers signals to induce apoptosis. We thus investigated the role of ER stress in BP-induced cell death in prostate cancer cells.

**Table 1:** Gene expressed differently in BP-treated LNCaP cells.

		Change* (∼fold)	
Functional Classification and Accession No	Representative Gene Name	24 h	3 h
**Signal transduction**			
NM 004864	NSAID-activated gene 1 (NAG-1)	5.66	2.92
**Transcription factor**			
NM 001924	Growth arrest and DNA-damage-inducible, alpha (GADD45A)	4.05	3.43
**ER stress**			
NM 001195053	DNA-damage-inducible transcript 3 (DDIT3/GADD153/CHOP)	11.89	7.26
NM 001433	Endoplasmic reticulum to nucleus signaling 1 (ERN1/IRE1)	2.51	N/A
NM 007348	Activating transcription factor 6 (ATF6)	2.31	N/A
**Cell cycle**			
NM 000389	Cyclin-dependent kinase inhibitor 1A (p21, Cip1)	6.29	N/A
**Apoptosis**			
NM 001225	Caspase 4	2.54	N/A
NM 006437	Poly (ADP-ribose) polymerase family, member 4 (PARP4)	3.414	N/A
NM 000043.4	Fas (TNF-receptor superfamily, member 6, CD95/APO-1)	2.191	N/A

* Genes list above are those that increased by >2-fold after BP treatment. N/A, Not Applicable.

### BP induced ER stress in LNCaP and PC-3 cells

To delineate the induction of ER stress by BP in prostate cancer cells, we investigated the induction of UPR related genes after BP treatment in LNCaP and PC-3 cells (Fig. 4A and B, respectively). Expression of BiP increased after BP treatment, but no differences were found in calnexin and PDI expressions (Fig. 4A and B). Up-regulation of ER stress transducer IRE1-α was observed but not ATF6 and p-eIF-2α. BP induced IRE1-α expression was evident from 3 h (1.16 folds) and increased till 48 h (2.47 folds) in LNCaP cells. The levels of Fas increased after BP treatment in both LNCaP and PC-3 cells and were consistent with the microarray finding in LNCaP cells. In addition, expressions of BiP, IRE1-α and GADD153 increased dose dependently in both LNCaP and PC-3 cells (Fig. 4C and D). The GADD153 transcriptional target, ER oxidase 1 like α (ERO1-Lα) also increased in a dose-dependent manner after BP treatment (Fig. 4C and D).

### BP-induced nuclear translocation of GADD153/CHOP in LNCaP and PC-3 cells

**Figure 5:**
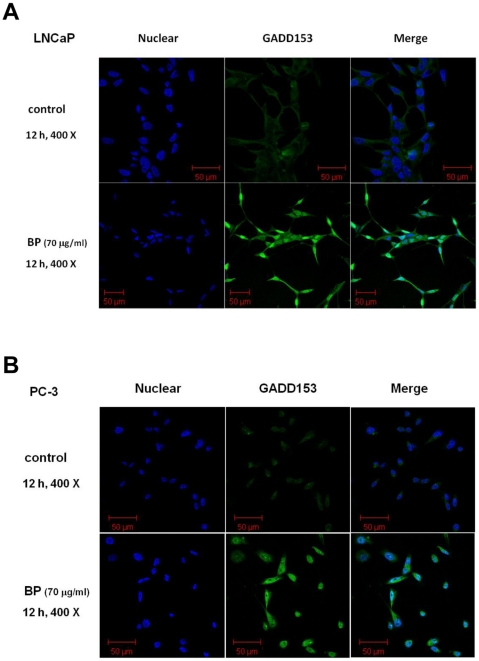
Nuclear translocation of GADD153/CHOP after BP treatment in human prostate cancer cells. (A) LNCaP cells and (B) PC-3 cells were treated with 0.2% DMSO (control) or 70 µg/ml BP for 12 h, fixed and stained with anti-GADD153/CHOP. FITC-labeled secondary antibody was used (green fluorescence) Nuclei were stained with DAPI (blue fluorescence). Images were captured by a confocal laser microscope.

To study the involvement of GADD153/CHOP in BP treatment, we first investigated whether BP promoted the translocation of GADD153/CHOP protein to the nucleus. GADD153/CHOP nuclear translocation represents the carrying of stress signal into the nucleus and therefore a functional activation. At 12 h after 70 µg/ml BP treatment, GADD153/CHOP was apparently more abundant in the nucleus of BP-treated LNCaP and PC-3 cells than in that of controls (Fig. 5A and B).

### The role of ER stress in BP-mediated anti-proliferation

**Figure 6:**
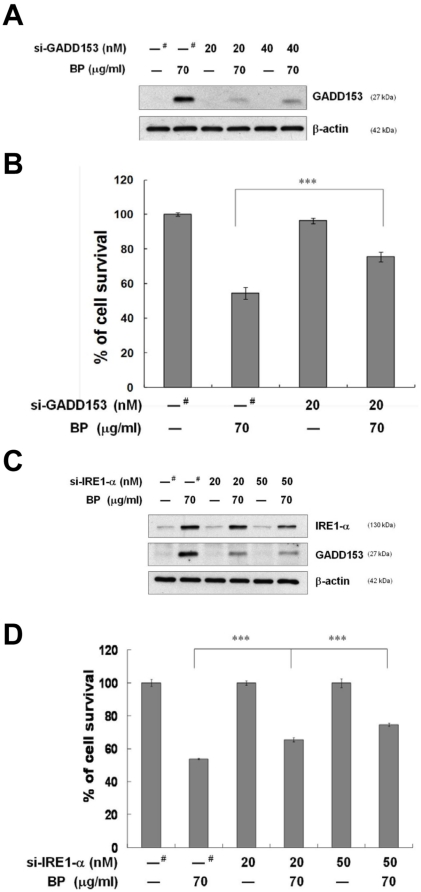
BP induces GADD153 and IRE1-α dependent cell death. (A) LNCaP cells were transfected with scramble siRNA (^#^), 20, or 40 nM GADD153 siRNA, respectively, for 48 h using the RNAifect transfection reagent. After 24 h treatment with 70 µg/ml BP, western blot analysis was performed for GADD153. (B) BP-induced anti-proliferative activity was measured with MTT assay in LNCaP cells transfected with scramble siRNA (^#^) or 20 nM GADD153 siRNA for 48 h and then treated with 70 µg/ml BP for 24 h. Data are expressed as means ± S.D. from three independent experiments. ***, *P*<0.001. (C) LNCaP cells were transfected with scramble siRNA (^#^), 20, or 50 nM IRE1-α siRNA, respectively, for 48 h using the RNAifect transfection reagent. After 24 h of treatment with 70 µg/ml BP, western blot analysis was performed for IRE1-α and GADD153, respectively. β-actin was used as an internal control. (D) BP-induced anti-proliferative activity was measured with MTT assay in LNCaP cells transfected with scramble siRNA (^#^), 20, or 50 nM IRE1-α siRNA, respectively, for 48 h and then treated with 70 µg/ml BP for 24 h. Data are expressed as means ± S.D. from three independent experiments. ***, *P*<0.001 versus vehicle.

We further used a siRNA approach to determine the role of GADD153 in the anticancer potential of BP in prostate cancer. LNCaP cells were transfected with siRNAs for GADD153 and IRE1-α, respectively, with or without post treatment of 70 µg/ml BP for 48 h. Western blot analysis demonstrated that transfection of si-GADD153/CHOP resulted in a suppression of GADD153/CHOP expression induced by BP in LNCaP cells, as compared with cells transfected with control scrambled siRNA (Fig. 6A). Cell death induced by BP treatment was rescued by 21.18% in cells transfected with GADD153/CHOP siRNA as compared to scrambled siRNA-transfected cells (Fig. 6B). Since GADD153/CHOP gene promoter contains binding sites for all of the major inducers of the UPR, and only IRE1-α was induced after BP treatment (Fig. 4), we further characterized the role of IRE1-α in BP-induced cell growth arrest and cell death. We silenced IRE1-α expression by si-IRE1-α transfection prior to 70 µg/ml BP treatment. As shown in Fig. 6C, IRE1-α siRNA significantly blocked IRE1-α protein expression induced by BP treatment in a dose dependent manner. In addition, GADD153/CHOP was also suppressed by IRE1-α siRNA transfection. MTT assay showed that 11.67 and 20.8% of cell death was inhibited by 20 and 50 nM IRE1-α siRNA transfection after exposure of cells to 70 µg/ml BP, respectively (Fig. 6D). Taken together, these results indicated that BP-induced cell death may be involved, at least in part, through the induction of ER stress and the up-regulation of IRE1-α and GADD153/CHOP proteins expression.

### The role of MAPK on BP-induced ER stress and cell death

**Figure 7:**
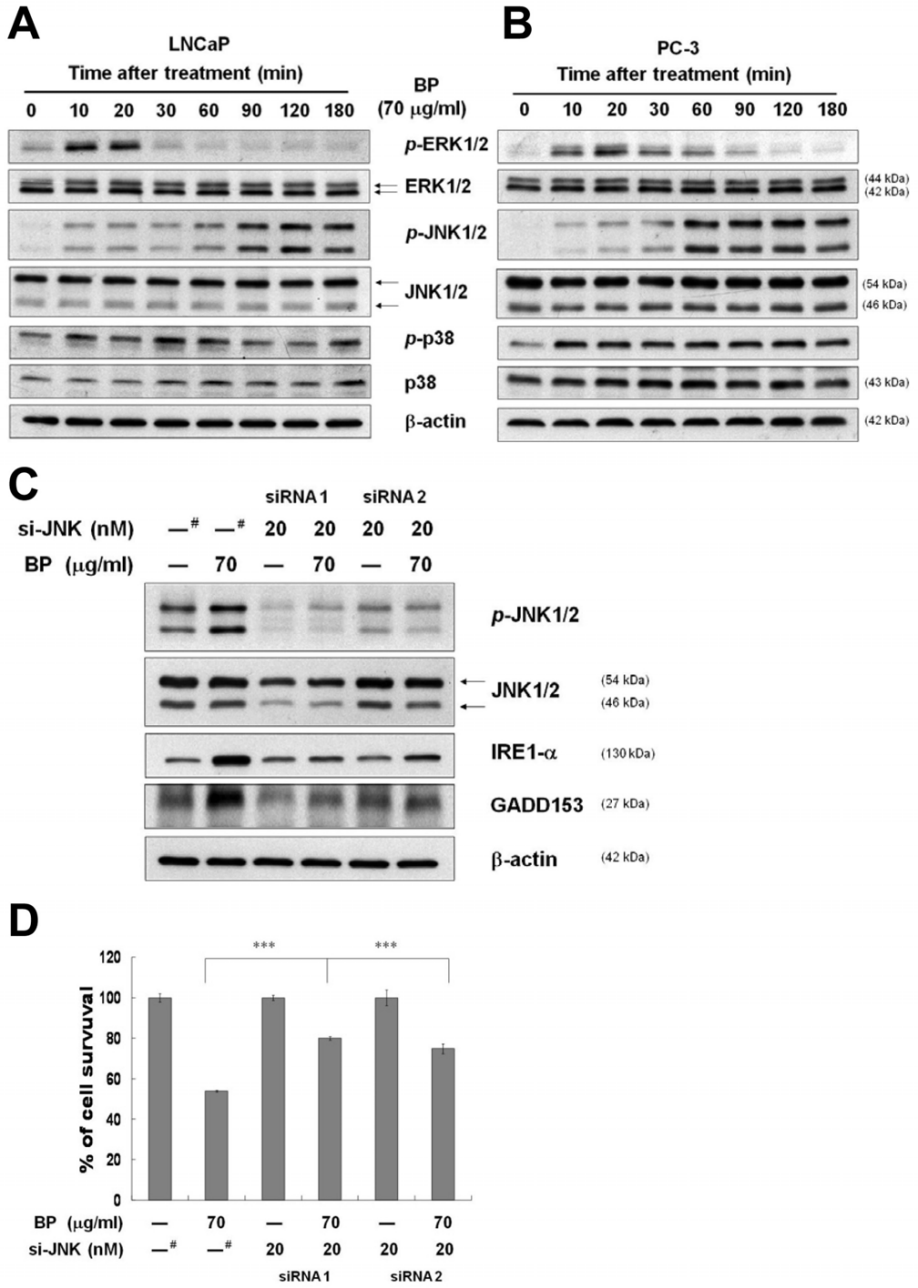
BP-induced ER stress and cell death are JNK1/2 dependent. (A) LNCaP cells and (B) PC-3 cells were treated with 70 µg/ml BP for the indicated times. Phospho-ERK1/2, total ERK1/2, phospho-JNK1/2, total JNK1/2, phospho-p38, and total p38 were detected by western blotting respectively. (C) LNCaP cells were transfected with scramble (^#^) or 20 nM JNK1/2 siRNA for 48 h using the RNAifect transfection reagent. After treatment with 70 µg/ml BP for 24 h, western blot analysis was performed for phospho-JNK1/2, total JNK1/2, IRE1-α and GADD153. β-actin was used as an internal control. (D) BP-induced anti-proliferative activity was measured with MTT assay in LNCaP cells transfected with scramble (^#^) or JNK1/2 siRNA for 48 h and then treated with 70 µg/ml BP for 24 h. Data was expressed as means ± S.D. from three independent experiments. ***, *P*<0.001 versus vehicle.

Activation of MAPKs has been implicated in the regulation of ER-stress induced cell death. We investigated the effect of BP on MAPK activation. Exposure of LNCaP and PC-3 cells to 70 µg/ml BP resulted in phosphorylation of JNK 1/2 and ERK 1/2, but not p38 MAPK (Fig. 7A and B). The ERK 1/2 phosphorylation after BP treatment occurred at 10 min and down-regulation occurred at 30 min. On the contrary, the phosphorylation of JNK 1/2 sustained at 10 to 180 min. To further characterize the role of JNK 1/2 in BP treatment, we silenced JNK 1/2 expression by specific siRNA transfection. As shown in Fig. 7C, JNK 1/2 expression and phosphorylation after BP treatment reduced by si-JNK transfection. In addition, the BP-induced expression of IRE1-α and GADD153 also reduced by si-JNK transfection. BP-induced cell death was rescued by si-JNK transfection, as compared to scrambled siRNA transfection (siRNA1: 26.14%, siRNA2: 20.95%, Fig. 7D). These findings suggest that JNK activation is involved in BP-induced cell death and participates in IRE1-α and GADD153 activation.

### BP inhibits growth of cancer cells in LNCaP–NOD-SCID xenograft

**Figure 8:**
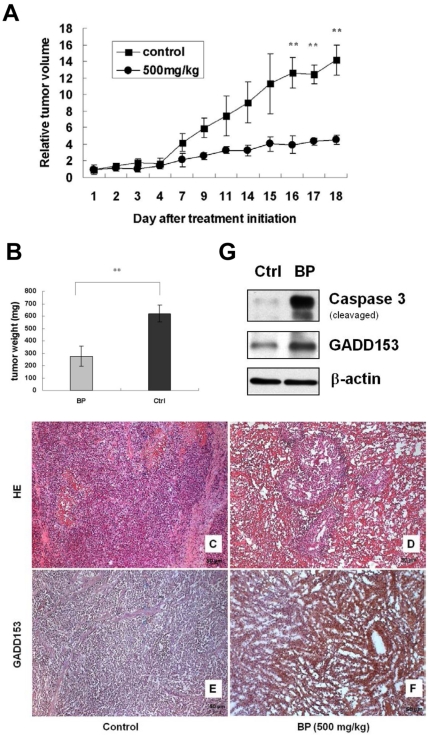
BP inhibits xenographic growth of LNCaP cells in NOD-SCID mice. (A) NOD-SCID mice were injected with approximately 5×10^5^ LNCaP cells into the dorsal subcutaneous tissue. When the tumor reached 100–250 mm^3^, LNCaP tumor-bearing mice were administrated s.c. with vehicle control (▪) or 500 mg/kg BP (•) on days 0–4 for 5 days. The relative tumor volumes of control and the BP-treated groups were shown as means ± S.D. of tumor volume at each time point. (B) Tumors of control and therapeutic groups were removed and weighted on day 18. Average tumor weight from the BP-treated group was 56% smaller than control group. Data was expressed as means ± S.D. of tumor weight of the control and the BP-treated groups. **, *P*<0.01. (C, D) Tumor tissue sections with HE staining of control (C) and therapeutic groups (D).GADD153 expressions were immunohistochemically identified in the control (E) and the BP-treated group (F). The GADD153-positive cells were stained brown. Expression of GADD153 and active form caspase-3 in the LNCaP xenograft tumor tissue were upregulated after BP administration as compared to control group by western blotting analysis (G).

To evaluate the antitumor activity of BP *in vivo*, human prostate cancer xenograft were established by subcutaneous injection of 5×10^5^ LNCaP cells into the dorsal subcutaneous tissue of NOD-SCID mice. As shown in Fig. 8A, the relative tumor volume in mice treated with 500 mg/kg BP was significantly 68% lower than the vehicle-treated control mice on day 18. Tumor weight was significantly decreased 56% in the BP-treated group as compared to the control group on day 18 (Fig. 8B). The tumors in control animals were graded as Gleason 5B since no glandular tissue was found (Fig. 8C). In BP-treated animals, irregular fused glands were observed and were staged Gleason 4A (Fig. 8D). Thus, the degrees of tumor differentiation of BP-treated group were better than the control group. Besides, up-regulation of GADD153/CHOP in the BP-treatment tumor was observed by immunohistochemistry staining (Fig. 8E and F) and western blot analysis (Fig. 8G), respectively. Caspase-3 activation was also observed in BP-treatment tumor (Fig. 8G). There were no significant differences of body weight in both control and BP-treated groups. These results indicated that BP-induced prostate cancer cell death was correlated with ER stress *in vivo*.

## Discussion

Currently some promising naturally-occuring dietary therapeutic compounds include resveratrol, curcumin, genistein, diallyl sulfide and other substances which have in common ability to induce apoptosis of cancer cells have been exploited [15]. In our previous studies, we demonstrated that BP act strongly against glioblastoma multiforme (GBM) brain tumors and hepatocellular carcinoma (HCC) tumors *in vitro* and *in vivo* [5], [6], [16]. However, the effects of BP on human prostate cancer cells have not been determined. Results from this study showed that BP is cytotoxic for the three different prostate cancer cell lines. Because both of the androgen-dependent and -independent prostate cancer cells showed similar sensitivity to BP treatment, the involvement of androgen pathway was ignored in this study.

The loss of cell cycle regulation is the hallmark of cancer. To explore the mechanisms accounted for the effect of BP on prostate cancer cells, cell cycles were monitored. BP-induced cell cycle arrest was evidenced by up-regulation of p16, p21 and p27, and down-regulation of CDK2 and cyclin D1. The p16, p21 and p27 proteins are cdk inhibitors that negatively regulate cdk or cyclin/cdk complex [17]. The p16 protein, member of the INK4 family, binds to cdk4/6 to block kinase activity at the mid-G1 phase [18]. The p21 (cip1) and p27 (kip1) proteins bind to the cyclin/cdk complex, resulting in the inhibition of G1 to S phase transition via the abrogation of Rb phosphorylation by the cyclin/cdk complex [19]. Thus, the decrease of phosphorylated Rb should be due to a BP-triggered expression of the cdk inhibitors, which in turn decrease the activity of the cyclin/cdk complex. Frequent activation of Akt signaling pathway has been reported in many human cancers [20], [21], and GSK-3β has been identified as one of the Akt's molecular targets. Akt inactivates GSK-3β kinase activity through site-specific phosphorylation at Ser9 [22]. Suppression of Akt which leads to de-phosphorylation and activation of GSK-3β cause phosphorylation of cyclin D1 at Thr286 and the subsequent proteasomal degradation [23]. Cyclin D1 has been shown to be overexpressed in many cancers including breast, head and neck, esophagus and prostate [24], . In this study, we found that BP is capable of inhibiting Akt activation by reducing Ser473 phosphorylation, and restores GSK-3β kinase activity by reducing Ser9 phosphorylation in prostate cancer cells. These data suggest that BP suppresses prostate cancer cells proliferation via cell cycle arrest by regulating the cyclin/CDK/CKI cell cycle regulatory protein and targeting the Akt-GSK-3β-Cyclin D1 signaling axis.

To investigate the underlying molecular mechanisms of BP-induced prostate cancer cells death, we examined the BP-induced changes in gene expression in human prostate cancer cells using an oligodeoxynucleotide-based microarray screening assay. Several genes showing greater than two-fold increase in expression at 24 h of BP treatment were identified (Table 1). GADD153/CHOP showed dramatically 11.89 folds increase of expression after BP treatment. GADD153/CHOP is a gene of interest as it is involved in ER stress-mediated apoptotic pathway. Activation of the UPR plays a protective role to cells under ER stress [26]. However, prolonged activation of UPR by excessive ER stress can convert its role to cytotoxic by activation of multiple apoptotic pathways in mammalian cells [27]. The ER stress transducer proteins ATF6, IRE1-α and PERK constitute the core stress regulator of the UPR, and transduce signals from the ER to the cytoplasm and nucleus after ER stress [28]–[30]. In our study, BP induced only IRE1-α activation but not ATF6 or p-eIF2α in prostate cancer cells. Increasing and sustaining expression of IRE1-α and its downstream target GADD153/CHOP after BP treatment were observed in time- and dose- dependent manner. One of the mechanisms implicated in GADD153/CHOP-mediated apoptosis is oxidative stress [31]. Oxidation of the ER lumen is induced by the GADD153/CHOP downstream target ERO1-Lα. ERO1-Lα has been speculated to hyperoxidize the ER lumen, and causes cytotoxic reactive oxygen species (ROS) production that leads to cell death. Another mechanism was associated with the role of Bax in GADD153/CHOP induced apoptosis. In ER-stress-induced cardiomyocyte aopotosis, Bax increase with ER stress in a GADD153/CHOP-dependent manner [32]. Bax and Bak also modulate UPR by a direct interaction with IRE1-α, and are important link between IRE1-α mediated ER-stress-induced apoptosis. Our results showed that transfection of siRNAs for IRE1-α or GADD153/CHOP suppressed BP-induced cell death. Taken together, these findings suggest that BP may modulate ER stress-mediated apoptosis of human prostate cancer cells through activation of IRE1-α-GADD153/CHOP signaling pathway.

Treatment with BP triggered increased expression of Fas, which led to the activation of caspase-8 and -3 in malignant brain tumor in our previous study [5]. Fas overexpression also increased at 3 h after BP treatment. Thus, BP-induced apoptosis of human prostate cancer cells might be mediated, at least partially, through death receptor apoptosis pathway.

Furthermore, activation of MAPKs has been implicated in the regulation of gene expression in the ER stress signaling cascade and is involved in many aspects of the control of cellular proliferation and apoptosis. The JNK pathway has also been shown to be a positive regulator of ER stress induced apoptosis [33]. In our study, phosphorylation of JNK was observed after BP treatment. Inhibition of JNK expression by siRNA led to the down-regulation of IRE1-α and GADD153/CHOP, and partially rescued BP-induced cell death. ER stress-activated IRE1-α binds the adaptor protein tumor necrosis factor receptor-associated factor2 (TRAF2), an E3 ubiquitin ligase, which in turn activates the apoptosis signal-regulating kinase 1 (ASK1; also known as MAP3K5), subsequently causes JNK activation. These findings implicate the notion that BP-induced ER stress-mediate cell death via the cooperation of the JNK pathway.

Tumor necrosis factor related apoptosis inducing ligand (TRAIL) is a death receptor ligand that can preferentially initiate apoptosis in various tumor cells. Recently, TRAIL-based therapeutics, including TRAIL gene therapy, recombinant TRAIL, and TRAIL-receptor-agonistic antibodies have entering clinical trials [34]–[37]. However, similar to many other cancers, prostate cancer develop resistance to TRAIL [38], [39]. Therefore, seeking for TRAIL sensitizers capable of overcoming TRAIL resistance in cancer cells are valuable. LNCaP are known to be resistant to TRAIL-induced apoptosis associated with the absence of PTEN that promote constitutive activation of the AKT/PI3K pathway. Since BP efficiently diminished the Akt activity by reducing the level of phospho-Akt Ser473, which makes BP a candidate of TRAIL sensitizer.

**Figure 9:**
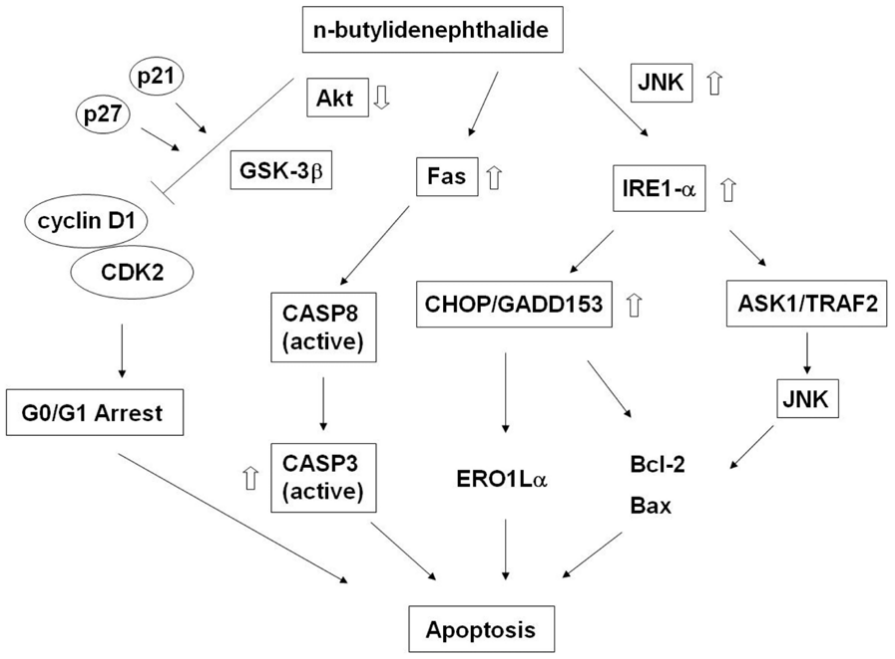
Schematic model of BP-induced apoptosis in human prostate cancer cells. BP induces apoptosis in human prostate cancer cells through multiple apoptotic pathways, including G0/G1 phase cell cycle arrest, death receptor pathway and ER stress-dependent pathway.

In conclusion, our study demonstrated that BP 1) causes prostate cancer cell cycle arrest at G0/G1 phase by targeting the Akt-GSK-3β-Cyclin D1 signaling axis; 2) induces cell death via ER-stress- and JNK-mediated UPR; 3) triggers apoptosis of human prostate cancer cells through multiple apoptotic pathways *in vitro* and *in vivo* (Fig. 9).

## Materials and Methods

### Cell cultures and Reagents

The human prostate cell lines LNCaP and PC-3 were purchased from ATCC (American Type Culture Collection, Manassas, VA), and cultured in RPMI 1640 medium with 10% heat-inactivated fetal bovine serum, 100 U/ml penicillin and 100 U/ml streptomycin, 1% sodium pyruvate, 2 mM L-glutamine (all of these reagents are from Invitrogen, Carlsbad, CA) at 37°C in a humidified atmosphere with 5% CO2. n-Butylidenephthalide (Lancaster Synthesis Ltd., Newgate, Morecambe, UK) was dissolved in dimethyl sulfoxide (DMSO; Sigma, St. Louis, MO, USA) to a concentration of 100 mg/ml and stored at −20°C as a master stock solution. The RNA isolation kit was purchased from QIAGEN (Valencia, CA). Dimethyl sulfoxide (DMSO), 3-(4,5-dimethyl thizol-2-yl)-2,5-diphenyl tetrazolium bromide (MTT), paclitaxel, and horseradish peroxidase-conjugated secondary antibodies were purchased from Sigma Chemical Co. (St. Louis, MO, USA). Polyvinyldenefluoride (PVDF) membranes, BSA protein assay kit and western blot chemiluminescence reagent were purchased from Amersham Biosciences (Arlington Heights, IL).

### Microarray analysis

A total of 100 ng of total RNA was amplified according to Affymetrix's small sample protocol (GeneChip Eukaryotic Small Sample Target Labeling Technical Note), and 15 µg of cRNA was then hybridized to Human gene 1.0 ST array (Affymetrix) and scanned. Image files were processed using MAS5.0 to produce Affymetrix expression data files. Data were then imported into GeneSpring (7.2; Silicon Genetics) and per chip normalization performed, using the 50th percentile of all measurements in that sample. All data is MIAME compliant and that the raw data has been deposited in a MIAME compliant database (accession number: GSE33883, http://www.ncbi.nlm.nih.gov/geo/query/acc.cgi?token=nropzqwssewqylo&acc=GSE33883)

### Western blot analysis

LNCaP cells were lysed on ice with 200 µl lysis buffer (50 mM Tris-HCl, pH 7.5, 0.5 M NaCl, 5 mM MgCl2, 0.5% Nonidet P-40, 1 mM phenylmethylsulfonyl fluoridefor, 1 µg/ml pepstatin, and 50 µg/ml leupeptin) and centrifuged at 13,000× g at 4°C for 5 min. The protein concentrations in the supernatants were quantified using a BSA Protein Assay Kit. Electrophoresis was performed on a NuPAGE Bis-Tris Electrophoresis System using 20 µg of reduced protein extract per lane. Resolved proteins were transferred to PVDF membranes, blocked with 5% non-fat milk for 1 h at room temperature, finally probed with appropriately dilution of primary antibodies at 4°C overnight: GADD153/CHOP, Bip, calnexin, PDI, IRE1-α, Ero1-Lα, cyclin D1, CDk2, phospho-Rb (Ser807/811), phospho-Akt (Ser473), Akt, phospho-GSK3β (Ser9), GSK3β, phosphor-ASK1 (Thr845), Fas, cleaved caspase-3 (Asp175), caspase-8 (1C12), Bax, MAPK family antibody, phosphor-MAPK family antibody, p16, p21 and p27 (Cell Signaling Technology, Inc., Danvers, MA). ATF6 (Abcam, Inc., Cambridge, MA), and ASK1 (GeneTex, Inc., San Antonio, TX). After the PVDF membrane was washed three times with TBS/0.2% Tween 20 at room temperature, it was incubated with appropriate secondary antibody labeled with horseradish peroxidase (goat anti-mouse or anti-rabbit, 1∶10000, Sigma Chemical, St. Louis, MO) for 1 h at room temperature. All resolved proteins bands were detected using Western Lightning™ Chemiluminescence Reagent Plus (Amersham Biosciences, Arlington Heights, IL) and quantified with densitometers.

### Growth inhibition assay

The viability of cells after treatment with various chemicals was evaluated using MTT assay preformed in triplicate. Briefly, the LNCaP cells (2×10^5^/well) and PC-3 cells (2×10^5^/well) were incubated in 6-well plates containing 2 ml of serum-containing medium. Cells were allowed to adhere for 18–24 h, and were washed with phosphate-buffered saline (PBS). Solutions were always freshly prepared by dissolving 0.2% DMSO (control) or drugs in serum-containing culture medium before their addition to LNCaP cells. The drug-containing medium was removed after treatment for indicated time, cells were washed with PBS, and culture medium containing 300 µg/ml MTT was added for 1 h at 37°C. After the MTT medium was removed, 2 ml of DMSO were added to each well. Absorbance at 570 nm was detected by a PowerWave X Microplate ELISA Reader (Bio-Tek Instruments, Winooski, VT). The absorbance for DMSO-treated cells was considered as 100%.

### Cell cycle analysis

The cell cycle was determined by flow cytometry using DNA staining dye to reveal the total amount of DNA. Approximately 5×10^5^ LNCaP cells were incubated with 70 µg/ml BP for the indicated time. Cells were harvested with trypsin/EDTA, collected, washed with PBS, fixed with cold 100% ethanol overnight, and then stained with a solution containing 20 µg/ml PI, 0.2 mg/ml RNase A, and 0.1% Triton X-100 for 30 min in the dark. The cells were then analyzed with FACScan flow cytometer (equipped with a 488-nm argon laser) to measure the DNA content. The data were obtained and analyzed with CellQuest 3.0.1 (Becton Dickinson, Franklin Lakes, NJ) and ModFitLT V2.0 software.

### Transfection with siRNA

GADD153/CHOP and IRE1-α siRNA were designed by siGENOME ON-TARGET plus SMARTpool siRNA purchased from Dhamarcon RNAi Technologies. GADD153/CHOP (DDIT3) target sequences are: GGUAUGAGGACCUGCAAGA, CACCAAGCAUGAACAAUUG, GGAAACAGAGUGGUCAUUC, CAGCUGAGUCAUUGCCUUU. IRE1-α (ERN1) targetsequences are: CUACCCAAACAUCGGGAAA, CUCCAGAGAUGCUGAGCGA, AUAAUGAAGGCCUGACGAA, GUCCAGCUGUUGCGAGAAU. Non-targeting control sequences were not provided. JNK 1/2 siRNA (1, # 6232, 2, # 6233) was purchased from Cell Signaling Technology, Inc. (Danvers, MA) and sequences were not provided. LNCaP cells at 50 to 60% confluence were transfect with siRNA (10–50 nM) using the RNAifect Transfection Regaent (QIAGEN) according to the manufacturer's protocol. Cells were cultured for 48 h, and then treated with BP or vehicle for an additional 24 or 48 h. Protein were then isolated for western blotting, or cells were collected for the MTT assay.

### TUNEL assay

LNCaP and PC-3 cells were cultured in the presence or absence of BP (70 µg/ml) for 48 h and then examined for apoptosis with TUNEL assay (In Situ Cell Death Detection kit, Roche).

### Immunocytochemistry

LNCaP cells cultured on glass slides were treated with 70 µg/ml BP for 12 h prior to fixation with cold 4% para-formaldehyde. The fixed cells were washed twice in PBS, and incubated in cold permeabilization solution (0.3% Triton X-100+0.1% sodium citrate). After endogenous peroxidase activity was inactivated with 3% H_2_O_2_, the cells were washed with PBS and incubated with an anti-GADD153 at 4°C overnight. The cells were washed with PBS three times and then incubated with FITC-conjugated secondary antibody 1 h at room temperature. The cells were then washed with PBS three times and stained with 300 nM DAPI for 10 min. Images were obtained with the confocal microscope (Carl Zeiss, Oberkochen, Germany).

### Animal studies

Ethics Statement: The animal use protocol listed below has been reviewed and approved by Institutional Animal Care and Use Committee (IACUC), Buddhist Tzu Chi General Hospital, approval No: 95-51.

To examine the anti-tumor effects of BP *in vivo*, the LNCaP human prostate cancer cells were used in male NOD-SCID mice experiments (8–10 weeks). The mice were purchased from the National Laboratory Animal Center (Taipei, Taiwan). All procedures were performed in compliance with the standard operating procedures of the Tzu Chi University Laboratory Animal Center (Hualien, Taiwan). All experiments were carried out using 6–8 week old mice weighing 18–22 g. The animals were subcutaneous implanted with 5×10^5^ LNCaP cells into the back of mice. When the tumor reached 80–150 mm^3^ in volume, animals were divided randomly into control and test groups consisting of six mice per group (day 0). Subcutaneous injection of either corn oil (control group), or 500 mg/kg of BP (treatment groups) was given for five successive days. BP was dissolved in a vehicle of 20% Tween 80 in normal saline (v/v). The injection sites were >1.5 cm from the tumors. Mice were weighed three times a week up to day 18–21. The tumor volume was also determined by measurement of the length (L) and width (W) of the tumor. The tumor volume at day n (TVn) was calculated as TV (mm^3^) = (L×W^2^)/2. The relative tumor volume at day n (RTVn) versus day 0 was expressed according to the following formula: RTVn = TVn/TV0. Tumor regression (T/C (%)) in treated versus control mice was calculated using: T/C (%) = (mean RTV of treated group)/(mean RTV of control group)×100. Xenograft tumors as well as other vital organs of treated and control mice were harvested and fixed in 4% formalin, embedded in paraffin, and cut in 4-mm sections for histological analysis.

### Statistical analysis

All data are shown as mean ± S.D. Statistical differences were analyzed using the Student's t-test for normally distributed values and by nonparametric Mann–Whitney *U*-test for values with a non-normal distribution. Values of *P*<0.05 were considered significant.
